# A transgenic cell line with inducible transcription for studying (CGG)n repeat expansion mechanisms

**DOI:** 10.18699/VJ21.014

**Published:** 2021-02

**Authors:** I.V. Grishchenko, A.A. Tulupov, Y.M. Rymareva, E.D. Petrovskiy, A.A. Savelov, A.M. Korostyshevskaya, Y.V. Maksimova, A.R. Shorina, E.M. Shitik, D.V. Yudkin

**Affiliations:** State Research Center of Virology and Biotechnology “Vector”, Rospotrebnadzor, Koltsovo, Novosibirsk region, Russia; International Tomography Center of Siberian Branch of the Russian Academy of Sciences, Novosibirsk, Russia Novosibirsk State University, Novosibirsk, Russia; International Tomography Center of Siberian Branch of the Russian Academy of Sciences, Novosibirsk, Russia; International Tomography Center of Siberian Branch of the Russian Academy of Sciences, Novosibirsk, Russia; International Tomography Center of Siberian Branch of the Russian Academy of Sciences, Novosibirsk, Russia; International Tomography Center of Siberian Branch of the Russian Academy of Sciences, Novosibirsk, Russia; Novosibirsk State Medical University, Novosibirsk, Russia Novosibirsk City Clinical Hospital No.1, Novosibirsk, Russia; Novosibirsk City Clinical Hospital No.1, Novosibirsk, Russia; State Research Center of Virology and Biotechnology “Vector”, Rospotrebnadzor, Koltsovo, Novosibirsk region, Russia; State Research Center of Virology and Biotechnology “Vector”, Rospotrebnadzor, Koltsovo, Novosibirsk region, Russia

**Keywords:** hereditary intellectual disability, fragile X syndrome, repeat expansion, transcription, replication, transgenic cell lines, somatic instability, наследственная умственная отсталость, синдром ломкой X-хромосомы, экспансия повторов, транскрипция, репликация, трансгенная клеточная линия, соматическая нестабильность

## Abstract

There are more than 30 inherited human disorders connected with repeat expansion (myotonic dystrophy type I, Huntington’s disease, Fragile X syndrome). Fragile X syndrome is the most common reason for inherited intellectual disability in the human population. The ways of the expansion development remain unclear. An important feature of expanded repeats is the ability to form stable alternative DNA secondary structures. There are hypotheses about the nature of repeat instability. It is proposed that these DNA secondary structures can block various stages of DNA metabolism processes, such as replication, repair and recombination and it is considered as the source of repeat instability. However, none of the hypotheses is fully confirmed or is the only valid one. Here, an experimental system for studying (CGG)n repeat expansion associated with transcription and TCR-NER is proposed. It is noteworthy that the aberrations of transcription are a poorly studied mechanism of (CGG)n instability. However, the proposed systems take into account the contribution of other processes of DNA metabolism and, therefore, the developed systems are universal and applicable for various studies. Transgenic cell lines carrying a repeat of normal or premutant length under the control of an inducible promoter were established and a method for repeat instability quantification was developed. One type of the cell lines contains an exogenous repeat integrated into the genome by the Sleeping Beauty transposon; in another cell line, the vector is maintained as an episome due to the SV40 origin of replication. These experimental systems can serve for finding the causes of instability and the development of therapeutic agents. In addition, a criterion was developed for the quantification of exogenous (CGG)n repeat instability in the transgenic cell lines’ genome.

## Abstract (ru)

Существует ряд наследственных заболеваний человека, причиной которых является экспансия тандемных повторов. К ним относятся миотоническая дистрофия первого типа, болезнь Хантингтона, заболевания, ассоциированные с ломкой Х-хромосомой. Синдром ломкой Х-хромосомы – наиболее распространенная причина наследственной умственной отсталости у человека. На сегодняшний день причины развития экспансии остаются неисследованными. Важная особенность протяженных повторов – их способность формировать альтернативные вторичные структуры ДНК. Существуют гипотезы, объясняющие природу нестабильности повторов, однако все они предполагают возникновение устойчивых вторичных структур ДНК на различных этапах клеточного цикла. Источником нестабильности считаются нарушения в различных процессах метаболизма ДНК (репликация, репарация и рекомбинация), вызванные образованием вторичных структур. Однако ни одна из гипотез до конца не подтверждена и, видимо, не является единственно верной. Вероятно, в различных типах клеток и на определенных стадиях клеточного цикла источником нестабильности выступает множество процессов. В настоящей работе мы предлагаем экспериментальную систему для изучения вклада транскрипции и ассоциированной с ней репарации в нестабильность повтора (CGG)n, поскольку это наименее изученный механизм возникновения нестабильности. Однако предложенные модели могут учитывать вклад и других процессов метаболизма ДНК, например репликации, что делает полученные системы универсальными и применимыми в разных исследованиях. Нами были созданы трансгенные клеточные линии, несущие повтор нормальной и премутантной длины под тетрациклин-индуцируемым промотором. Один тип линий содержит плазмиду с экзогенным повтором, интегрированным в геном посредством транспозона Sleeping Beauty, в другой клеточной линии вектор поддерживается в виде эписомы благодаря ориджину репликации SV40. Такие трансгенные клеточные линии могут служить экспериментальной системой для поиска причин нестабильности и создания терапевтических средств. Кроме того, был разработан критерий для оценки нестабильности экзогенного (CGG)n повтора в геноме трансгенных клеточных линий, расчет которого не зависит от эффективности синтеза протяженных повторов.

## Introduction

Repeat expansion is a unique type of mutation that is characterized by a dramatic increase of the number of triplet repeats in DNA. Triplet repeats are more prone to expansion: to date, more than 30 diseases associated with their instability are known (Grishchenko et al., 2020). Fragile X syndrome, as the most common form of hereditary intellectual disability is also based on triplet repeat expansion. The cause of the disease is the expansion of the CGG repeat located in the 5′-untranslated region of the FMR1 gene. Normally, the repeats number is relatively stable and does not exceed 54 triplets; if the (CGG)n expansion increases up to 200 triplets, the FMR1 allele becomes premutant, and the ataxia/tremor syndromes and primary ovarian insufficiency syndrome associated with a Fragile X syndrome develop. The premutant allele frequency in the population is 1 : 100. Even though the clinical manifestations are often not observed, the expanded repeat can be transmitted over generations. Full mutation develops when triplets numbers increase over 200: the FMR1 gene promoter becomes methylated, the locus is heterochromatinized, and the FMRP protein is completely lost, which leads to the development of Fragile X syndrome. FMRP is necessary for normal neuron activity and its absence causes pronounced phenotypic manifestations: macroorchidism, endocrine pathologies, cerebellum morphological changes, and intellectual disability characterized by behavior and learning problems (Roberts et al., 2003; Martin et al., 2012; Heulens et al., 2013). The full mutation frequency varies from 1 : 4,000 in men, and up to 1 : 6,000 in women

Despite understanding the syndrome pathogenesis details, the expansion mechanism has not yet been studied. Different processes of DNA metabolism are probably able to increase the CGG-repeat instability. Therefore, the contribution of replication to the expansion processes has been established: the formed hairpin on the newly synthesized DNA strand leads to the additional replication of the region containing the (CGG)n repeat and, therefore, to its increasing (Fouche et al., 2006). However, in people suffering from repeat expansion disorders, and in model mice, expansion is also observed in tissues with low proliferative activity, including the brain lobes, oocytes, liver and muscles (Lokanga et al., 2013); it confirms the theory that the repeat expansion can also depend on other processes affecting DNA. Indeed, for many proteins of the DNA repair and recombination pathways, their probable participation in the repeat expansion process has been shown. Some experimental data indicate the MMR system components involvement in the expansion (Kovalenko et al., 2012; Zhao et al., 2016). Another possible source of instability can be transcription and transcription-coupled repair (TCR-NER), since many repeat tracts are characterized by the R-loops formation – RNA: DNA-resistant duplexes forming during RNA synthesis as well as the disruption of the initiation of PolII transcription (Krasilnikova et al., 2007). The lesions during transcription initiate TCR, a form of excisional nucleotide repair (NER). For some proteins of this cascade, correlations with the (CGG)n instability level were found. Itshould be noted that for the FMR1 premutant alleles, which rapidly accumulate repeated units, a significant increase of the FMR1 transcription level was found, which probably indicates the involvement of the TCR system in the repeat instability development. However, there is no unequivocal confirmation of this hypothesis.

To study the details of all the described cascades, it is necessary to have a model in which it is possible to track all the changes occurring with repeat and surrounding regions in response to the induction of a certain DNA metabolism process. To date, similar models have already been proposed (Gorbunova et al., 2003; Kononenko et al., 2020), but none of them can directly assess the contribution of transcription to (CGG)n instability. In this study, we describe the experimental models for repeat instability research based on two types of plasmids: integrated and not integrated into the genome. These models will allow taking into account the contribution of replication, transcription, TCR-NER, and genome location to the CGG-repeat instability. In addition, this model can be used to study the repeat-induced mutagenesis observed in cells with an expanded repeat in the FMR1 promoter region (Shah, Mirkin, 2015).

## Materials and methods

**Ethics statements.** The procedure of involving the patients in the study was strictly designed in accordance with international standards, which include the awareness of the subject, their consent to participate in the study in its entirety, and the guarantee of confidentiality. All of the studies conformed to the ethical standards developed in accordance with the Helsinki Declaration of the World Medical Association as amended in 2000. In addition, the studies were supervised by the Institutional Review Board. The written consent of the study participants was also obtained.

**DNA purification and repeat sizing.** Peripheral venous blood from all of the patients was collected in Novosibirsk City Clinical Hospital No. 1 into EDTA-containing tubes and frozen before DNA purification. The DNA was purified from whole venous blood and cell cultures using a Wizard® Genomic DNA Purification Kit (Promega, USA). 

CGG repeats were sized using a special protocol for the GC-rich DNA amplification proposed earlier (Hayward et al., 2016). For PCR primers NewFraxC (5′-d6RG-tgctttc tagactcagctccgtttcggtttcacttccggt-3′) and NewFraxR4 (5′-taa gcagaattcccttgtagaaagcgccattggagccccgca-3′) and 0.02 units of Q5-DNA polymerase were used. The resulting fragments was separated by agarose gel electrophoresis. To assess the accurate size of the repeat, capillary electrophoresis using a 1200 LIZ length standard (AppliedBiosystems, USA) was performed. The flanking region in the PCR product is a total of 269 bp, thereby the repeat length was determined by the following equation

**Formula:**
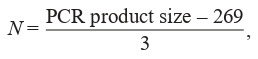
(1) where N – CGG-triplets number

**Cloning CGG repeats of various lengths into vector systems.** The control plasmid pCDH containing no CGG-repeat consisted of the following elements: (1) doxycyclineinducible Tet-O-minimal CMV promoter, IRES sequence, open reading frame (ORF) of the GFP protein, (2) constitutive promoter EF1alpha, transactivator for Tet-O-element rTtA ORF, T2A peptide, the DsRedExpress protein ORF, (3) beta-lactamase promoter, beta-lactamase protein ORF for transformed bacterial cells selection, origin of replication, and (4) SV40 origin of replication. The PCR product carrying the CGG repeat was cloned into the pCDH plasmid at the XbaI and EcoRI restriction endonuclease sites (SibEnzyme, Russia) between the CMV minimal promoter and the IRES sequence.

Plasmid pSBi for CGG-repeat cloning was assembled from the following components: (1) beta-lactamase promoter, betalactamase protein ORF for transformed bacterial cells selection, origin of replication, (2) Sleeping Beauty transposon terminal repeats, (3) cassette containing a PGK promoter and a puromycin-N-acetyl transferase ORF, (4) an hPGK promoter, an rTta ORF, (5) an inducible TRE3GS promoter, and an mGFP ORF. 

Cloning of the PCR product containing CGG-repeat driven by an inducible promoter was also carried out at the restriction endonuclease sites XbaI and EcoRI (SibEnzyme). For the plasmids production, the electrocompetent cells of the E. coli strain NebStable (NEB, USA) were transformed. It was shown that an extended repeat during the transformation of bacterial cells and their cultivation is prone to a dramatically repeat length contraction, which is consistent with the literature data (Bontekoe, 2001); therefore, the NebStable cells were cultured for a day at 20 °C to avoid the repeat size decrease. For HEK293A and HEK293T cells transfection, plasmids were isolated and purified using the QIAGEN® Plasmid Plus Maxi Kit (QIAGEN, Germany).

**Eukaryotic cell transfection.** HEK293A and HEK293T cells transfection was performed using the Lipofectamine 3000 reagent (Thermo Fisher Scientific, USA). The induction of the Tet-O-minimal CMV promoter and TRE3GS promoter was performed using the doxycycline with a concentration of 1 μg/ml in the cultural media.

## Results

**Assembly of experimental plasmids carrying CGG-repeat of normal and premutant lengths**

**Fig. 1.**
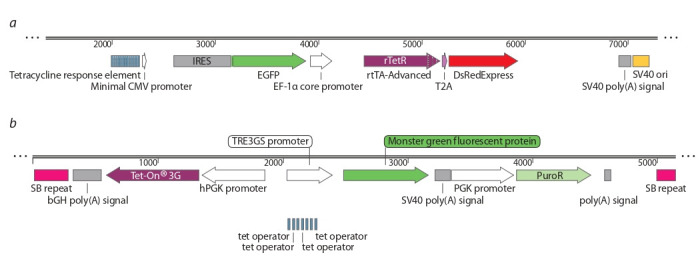
Vector maps used to generate model cell lines а – pCDH plasmid map. IRES – internal ribosome entry site; EGFP – green fluorescent protein ORF; EF1 – constitutive promoter EF1alpha; rtTA – tetracycline/ doxycycline-interacting transactivator for tetracycline response element; DsRedExpress – red fluorescent protein ORF; SV40 ori – SV40 viral origin of replication; b – pSBi plasmid map. SB repeat – repeat that is recognized by the Sleeping Beauty transposase; Tet-On® 3G – tetracycline/doxycycline-interacting transactivator for TRE3GS promoter; hPGK – constitutive promoter; TRE3GS – inducible promoter; PGK – constitutive promoter; PuroR – puromycin-N-acetyl transferase ORF.

We obtained a set of plasmids based on eukaryotic expression vectors with an inducible promoter that regulates the CGG-repeat transcription level of CGG repeat of normal or premutant length and GFP ORF. These plasmids serve as the core of the model system for studying (CGG)n repeat instability. The pCDH plasmid was used as a vector for transient expression and exogenous CGG-repeat maintenance in a nonintegrated state in the genome (Fig. 1, a). For the integration of the exogenous CGG-repeat into the genome, a construct based on the Sleeping Beauty pSBi transposon/transposase system was assembled (see Fig. 1, b).

The pCDH plasmid encodes two reporter proteins: DsRedExpress driven by EF1 promoter and EGFP, whose expression is regulated by the inducible Tet-O-CMV promoter. Downstream of the Tet-O-CMV promoter, a multiple cloning site for CGG-repeat cloning is located. Due to this mutual arrangement of the inducible promoter and the site of the repeat cloning, it can be established that transcription goes through the inserted CGG-repeat due to the synthesis of EGFP mRNA. After several transcription rounds, the influence of transcription on the repeat instability can be detected. In addition, pCDH plasmid contains the SV40 origin of replication and thereby it is able to replicate in HEK293T cells that produce the SV40 large T antigen. In this case, it is possible to assess not only the contribution of transcription, but also the role of replication processes during the maintenance of the pCDH in the form of an episome.

The pSBi vector encodes an mGFP protein driven by an inducible TRE3GS promoter. Before the mGFP ORF are sites for cloning the CGG repeat. Therefore, it is possible to analyze the effect of transcription on changes in the CGG repeat length. Since this vector is based on a transposon, a part of the plasmid flanked by specific repeated sequences recognized by SB transposase and this part of plasmid can be inserted into different regions of the genome by transposase. It is possible to assess the potential influence of the integration sites on the instability of the CGG repeat by the determination of the insertion sites.

**Fig. 2.**
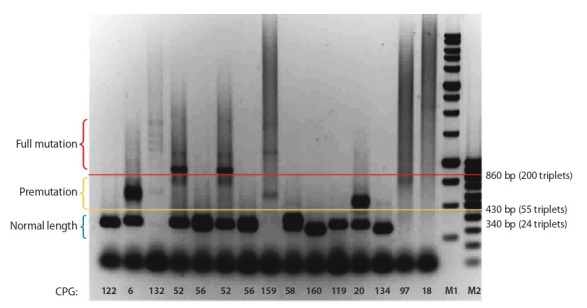
Sample of CGG-repeat amplification. CPG – samples of DNA from patients; M1 –1 kb DNA ladder; M2 – 100 bp DNA ladder.

To obtain fragments containing a CGG-repeat, we used DNA samples isolated from continuous human B-lymphocytes cultures and whole blood samples of patients with Fragile X syndrome (Fig. 2).

To create a construct with exogenous CGG-repeat it was decided to use repeats of normal and premutant lengths. It is expected that the instability of these types of repeats will be significantly different, since the premutant allele is the most unstable, and the normal allele, on the contrary, is prone to only insignificant polymorphism (Lokanga et al., 2013).

**Fig. 3.**
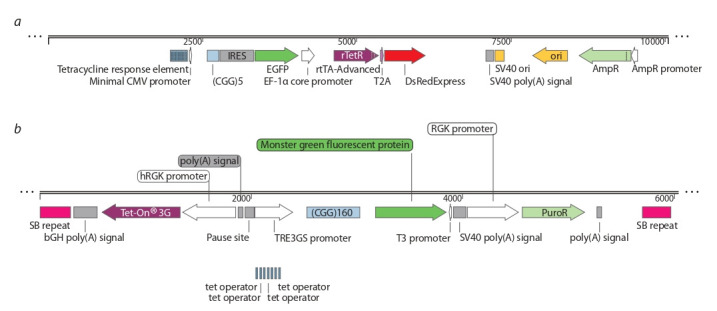
Maps of plasmids with cloned CGG repeat a – pCDH plasmid with CGG-repeat and b – pSBi plasmid with CGG-repeat.

As a result, five types of plasmids were obtained. These plasmids carry 5 (pCDH-5), 25 (pSBi-25), 59 (pCDH59), 85 (pCDH85), and 160 repeats (pSBi-160), respectively. The structures of all plasmids were confirmed by Sanger sequencing (Fig. 3).

**Study of the experimental plasmid functionality**

The eukaryotic cells transfection efficiency by the assembled constructs was evaluated to confirm the correct expression of reporter proteins in the presence of an extended repeat (CGG)n. It has been shown that transfection and reporter protein synthesis after transfections by plasmids carrying CGGrepeat of normal or premutant length occurs with the same efficiency as transfection with control plasmids (without the repeated sequence). When cells were transfected with pCDH plasmids, the expression of DsRedExpress was observed. It was also possible to carry out selection on a medium with the puromycin of cells transfected with pSBi vectors. The ability of the tetracycline/doxycycline-inducible promoters regulating green fluorescent protein expression to spontaneous activation was investigated. It is important that the transgenic cells do not have a background EGFP expression because spontaneous promoter activation can interfere with the accurate assessment of the CGG-repeat instability level during transcription and transcription-coupled repair 

**Fig. 4.**
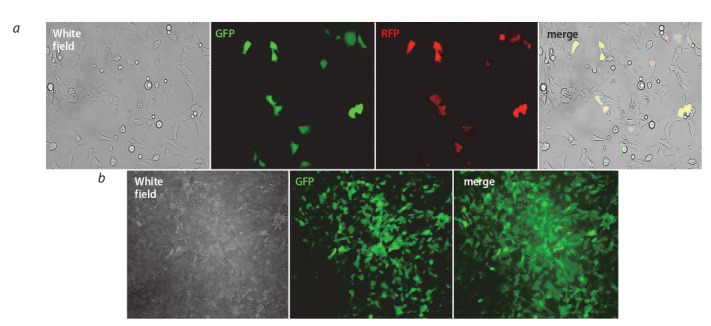
Induction of tetracycline-dependent promoters in the designed plasmids a – induction of Tet-O-promoter in HEK293T cells transfected by Pcdh; b – induction of TRE3GS in HEK293A cells transfected by pSBi and selected on puromycin

Cells transfected with pCDH the showed active expression of the red protein (driven by constitutive promoter EF1) and the absence of the green protein expression (regulated by inducible promoter) without the promoter induction. For induction, doxycycline was added daily to the cells, resulting in a high level of green protein fluorescence (Fig. 4, a). When plasmid pSBi containing the TRE3GS promoter was used, no background induction was observed. It allows for performing a selection using puromycin to obtain stable transformants and to avoid the background transcription level influence on the inserted CGG-repeat (see Fig. 4, b).

**Development of the method for analyzing repeat instability in model cell lines**

By using the obtained transgenic cell lines, we expect that the expansion in different cells of the culture will occur at different rates, and, as a result, we will receive a mosaic culture. In this regard, it is necessary to use a value allowing the grading of somatic instability and allowing the comparison of cell lines carrying different alleles of exogenous CGG-repeat. Previously, different approaches were proposed to assess the trinucleotide repeats somatic instability in patients with repeats expansion disorders. For example, the method for the assessment of CAG-repeat instability in Huntington’s disease is based on the main allele determination by the maximum peak as a result of fragment analysis and additional peaks, followed by normalization to the summed values of the heights of all peaks (Lee et al., 2010). Another method for the repeat instability level assessment is based on the serial dilution of the template followed by PCR – small-pool PCR (Monckton et al., 1995; Morales et al., 2012).

Amplification by dilution enables the detection of mosaicism, which cannot be detected by conventional PCR due to the low synthesis efficiency of less represented or very large alleles. However, these methods are insufficiently applicable to assess the instability (CGG)n, since the amplification of the larger allele occurs with much less efficiency than the shorter allele synthesis (Usdin, Woodford, 1995; Woodford et al., 1995; Jensen et al., 2010). To quantify the CGG-repeat instability in developed cell lines, we proposed an analysis method based on the calculation of the somatic instability index (ISI) after the amplification of GC-rich templates according to the method of B.E. Hayward et al. (2016). This value enables one to take into account not only the repeat size but also the spread of values between alleles, regardless of the efficiency of their synthesis. For (CGG)n repeats located in the FMR1 gene, we propose the calculation of ISI using the following equation

**Formula:**
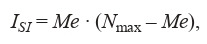
(2) where Me – median and Nmax – maximal length of CGG repeats in any sample

**Fig. 5.**
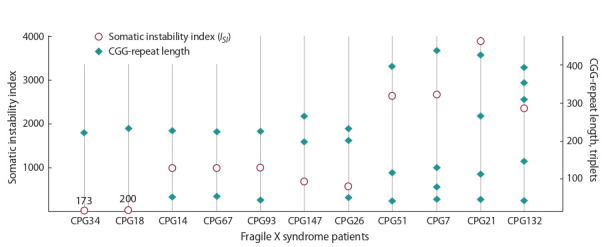
Repeat length and indexes of somatic instability in FXS patients. The values on the graph above the markers are the somatic instability indexes

The median is a value separating the raw data into two halves, and it considers the number of alleles. This value takes into account sample heterogeneity, and not sensitive to the detection of repeat lengths that are too long or too short, unlike using an arithmetic mean. When using the arithmetic mean in the index calculating, the contribution of larger alleles will be taken into account more than the contribution of shorter ones. As a result, cell lines with different degrees of the exogenous repeat instability can have similar values of the magnitude of somatic instability, which will lead to the false results interpretation. The value (Nmax – Me) takes into account the diversity and scatter of values in samples, where large Me values indicate a large median repeat length. The ISI calculation does not take into account the amount of PCR product (peak height) for each allele, i. e. PCR efficiency does not affect the final value. To determine the somatic instability index, the DNA of eleven patients with Fragile X syndrome was isolated from whole blood, which served as a starting material for the synthesis of extended repeats (CGG)n (Fig. 5).

As can be seen from the calculation of the ISI , index increases with an increase in the number and spread of repeat values. It should be noted that the method of analysis of instability works for two or more alleles in patients with mosaicism. In the case of one allele, we take the index of somatic instability equal to the size of the CGG-repeat, since the patient with one allele has Nmax – Me = 0. We cannot accept ISI = 0 because the CGG-repeat is unstable by nature.

## Discussion

Fragile X syndrome is one of the most common causes of hereditary intellectual disability (Yudkin et al., 2015). The frequency of full mutation in the human population varies from 1 : 6,000 in women to 1 : 4,000 in men, while the premutant allele, as the most unstable allele of the FMR1 gene promoter region, occurs in 1 : 100 cases. The instability of the CGG repeat is expressed in its tendency toward expansion – a multiple and rapid increase of the tract repeated sequence length. In addition, in the cells and tissues of patients as well as the tissues of model animals, repeat contractions are observed, that lead to somatic mosaicism and its degree correlates with the severity of symptoms (Mailick et al., 2018). However, the probability of expansion is in tenfold higher than contraction (Bontekoe, 2001; DeJesus-Hernandez et al., 2011), which may be the reason for the increased severity of the diseases manifestations during transmission in a number of generations

There are a number of hypotheses explaining the expansion mechanism, but none of them have been sufficiently supported by experimental data. All of the hypotheses – assume as a main reason for repeat instability – the formation of alternative DNA secondary structures at a certain site in DNA during the different processes of DNA metabolism, which can disrupt these processes. In vitro and in vivo experiments have shown the formation of alternative DNA secondary structures, such as hairpins, R-loops, and G-quadruplexes (Usdin, Woodford, 1995; Groh et al., 2014; Lam et al., 2014). Such structures can significantly violate these processes of DNA metabolism, which in turn affects the instability of repeats. One of the possible reasons is associated with the slippage of a DNA strand during replication (Pearson, Sinden, 1996; Fouche et al., 2006). Today, it is absolutely clear that the slippage of DNA strands can occur in various cases: during DNA replication in dividing cells as well as during repair processes. However, this model cannot reliably explain why not all of the repeats expand or and why the threshold value for the length of the repeat sequence is similar for different diseases. There is evidence for the contribution of some repair cascade proteins, which are proteins that are necessary for recombination and transcription to repeat instability. However, all of the hypotheses have certain drawbacks and contradictions; therefore, it is necessary to continue the search for the molecular mechanism of repeat expansion.

An expansion model based on a transgenic cell line containing exogenous (CGG)n repeat can serve as a convenient experimental system. In such systems it is possible to track changes in repeat length in response to the induction of different cascades of DNA metabolism. Using the transgenic cell lines obtained in this study make it possible to assess the contribution of replication, transcription, and repair in the cell to CGG-repeat instability. We have assembled two types of plasmids: based on the SV40 origin plasmid, capable of replicating in cell cultures expressing the SV40 T antigen, and based on the Sleeping Beauty transposon-based vector system for integrating the cassette with CGG-repeat and reporter proteins into various genomic loci. The transfection efficiency and the initial expression level of reporter proteins were comparable to those of the control plasmid without the (CGG)n repeat. It is also possible to obtain a transgenic cell culture with single genotype using different approaches such as sorting or limiting dilutions with antibiotic selection. Changes in the length of an exogenous repeat and, therefore, mosaicism that will take place in transgenic culture over time can be detected and estimated using the developed ISI index. This method is useable and reflects the correlation between repeat instability and phenotypic manifestations of the diseases observed in the different brain lobes of patients with Fragile X syndrome and associated disorders.

In the created experimental cell lines it is possible to directly assess the level of repeat expansion or contraction as well as the changes caused by repeat instability. The design of vector systems makes it possible to detect changes in the length of the exogenous CGG-repeat at different genome loci, during cultivation for a long or short time, with or without promoter induction. Measuring the fluorescent proteins expression levels can serve as basis for tracking the possible increase of instability and mutations accumulation mediated by repeatinduced mutagenesis. To determine the contribution of specific proteins from various cascades to the development of instability, it is possible to carry out chromatin immunoprecipitation using transformed cells. In addition, the level of instability in the created cellular models of CGG-repeat expansion can be assessed by the proposed index of somatic instability. Index ISI should also have a biological meaning, i. e. reflect the degree of phenotypic changes in patients with Fragile X associated disorders. To test this hypothesis, a study of the dependences of ISI values in patients with changes in the brain according to FMRI data was started. The preliminary data indicate some correlations, but more research is needed.

## Conclusion

To date, the mechanism of the instability of trinucleotide repeats remains not fully understood. At the same time, this research area remains extremely urgent due to the fact that the diseases caused by this mutation are socially significant. To search for the repeat instability reasons, it is necessary to develop cellular models for tracking all of the changes caused by expansion, as well as to evaluate the contribution of various proteins and DNA metabolism pathways to this process. The constructs developed in this work for instability assessing can be used in such studies. 

Various cell lines can be transfected with the assembled plasmids. We tested the efficiency of the constructs in two cell lines: HEK293A and HEK293T. After cell transfection and the induction of reporter protein expression, at various passages, it is possible to accurately determine the repeat size (CGG)n, as well as other parameters and show the presence or absence of CGG-repeat expansion, depending on its initial length and the number of passages. In the future, our model can be used in studies for the determination of all the aspects of repeat instability in the human genome and it will help form a more complete understanding of the mechanisms of this mutation.

## Conflict of interest

The authors declare no conflict of interest.
